# Development and Qualitative Evaluation of a Decision Support Tool for Withdrawal of Biologic Therapy in Nonsystemic Juvenile Idiopathic Arthritis

**DOI:** 10.1177/23814683251364199

**Published:** 2025-09-29

**Authors:** Janine A. van Til, Michelle M. A. Kip, Robert Marinescu-Muster, Karin Groothuis-Oudshoorn, Gillian R. Currie, Susanne M. Benseler, Joost F. Swart, Sebastiaan J. Vastert, Nico Wulffraat, Rae S. M. Yeung, Deborah A. Marshall, Maarten J. IJzerman

**Affiliations:** Department of Health Technology and Services Research, Faculty of Behavioural, Management and Social Sciences, Technical Medical Centre, University of Twente, Enschede, The Netherlands; Department of Health Technology and Services Research, Faculty of Behavioural, Management and Social Sciences, Technical Medical Centre, University of Twente, Enschede, The Netherlands; Department of Communication Science, Faculty of Behavioural, Management and Social Sciences, Technical Medical Centre, University of Twente, Enschede, The Netherlands; Department of Health Technology and Services Research, Faculty of Behavioural, Management and Social Sciences, Technical Medical Centre, University of Twente, Enschede, The Netherlands; Alberta Children’s Hospital Research Institute, University of Calgary, Calgary, AB, Canada; Department of Community Health Sciences, Cumming School of Medicine, University of Calgary, Calgary, AB, Canada; Department of Paediatrics, Cumming School of Medicine, University of Calgary, Calgary, AB, Canada; Alberta Children’s Hospital Research Institute, University of Calgary, Calgary, AB, Canada; Rheumatology, Department of Pediatrics, Alberta Children’s Hospital, Cumming School of Medicine, University of Calgary, Calgary, AB, Canada; Children’s Health Ireland, Dublin, Ireland; Department of Pediatric Rheumatology, Division of Paediatrics, University Medical Center Utrecht, Wilhelmina Children’s Hospital, Utrecht, The Netherlands; Faculty of Medicine, Utrecht University, Utrecht, The Netherlands; European Reference Network RITA (rare immunodeficiency autoinflammatory and autoimmune diseases network); Department of Pediatric Rheumatology, Division of Paediatrics, University Medical Center Utrecht, Wilhelmina Children’s Hospital, Utrecht, The Netherlands; Faculty of Medicine, Utrecht University, Utrecht, The Netherlands; European Reference Network RITA (rare immunodeficiency autoinflammatory and autoimmune diseases network); Department of Pediatric Rheumatology, Division of Paediatrics, University Medical Center Utrecht, Wilhelmina Children’s Hospital, Utrecht, The Netherlands; Faculty of Medicine, Utrecht University, Utrecht, The Netherlands; European Reference Network RITA (rare immunodeficiency autoinflammatory and autoimmune diseases network); Division of Rheumatology, The Hospital for Sick Children, Department of Paediatrics, Immunology and Institute of Medical Science, University of Toronto, Toronto, ON, Canada; Department of Community Health Sciences, Cumming School of Medicine, University of Calgary, Calgary, AB, Canada; Department of Medicine, Cumming School of Medicine, University of Calgary, Calgary, AB, Canada; Department of Health Technology and Services Research, Faculty of Behavioural, Management and Social Sciences, Technical Medical Centre, University of Twente, Enschede, The Netherlands; Erasmus School of Health Policy & Management, Erasmus University Rotterdam, the Netherlands

**Keywords:** juvenile idiopathic arthritis, biologicals, treatment withdrawal, decision support tool

## Abstract

**Introduction.** Limited evidence guides pediatric rheumatologists on when to withdraw biologic therapy in children with juvenile idiopathic arthritis, resulting in wide variation in clinical practice. This study aimed to develop and evaluate a decision support tool (DST) based on expert opinion to support pediatric rheumatologists in making withdrawal decisions. **Methods.** A literature review, focus groups, interviews, and prior research informed the design of the prototype DST. Evaluation of the DST’s face validity, content validity, acceptance, and feasibility was conducted through user testing interviews and a survey among pediatric rheumatologists from the Netherlands and Canada. Findings were summarized using descriptive and qualitative content analyses. **Results.** The prototype DST requires input on relevant patient, disease, and treatment characteristics. Its primary output is the predicted likelihood of biologic therapy withdrawal. Pediatric rheumatologists can adjust the importance of characteristics and observe the resulting impact on withdrawal likelihood. Eleven pediatric rheumatologists participated in testing. Key themes identified included the need for 1) clear terminology to ensure consistent interpretation of model inputs, 2) concise instructions on how and when to adjust the relative importance of characteristics, and 3) practice rounds to build trust among pediatric rheumatologists in the DST’s output. Participants found the DST feasible for clinical use, with its main value in explaining decisions to patients and engaging them in the decision-making process. Suggested future improvements include tracking the outcomes of withdrawal decisions and integrating predictive models based on clinical data. **Conclusions.** The DST developed in this study was well-received. Its main value lies in helping pediatric rheumatologists explain their decisions to patients and parents. The top priority for further development is integrating scientific evidence on successful withdrawal decisions.

**Highlights:**

- Decision support tools that provide structure to decisions based on expert opinion can increase transparency and consistency in medical decision making in the absence of clinical evidence.
- Data from clinical vignette studies that use an experimental design to elicit treatment preferences can be used to predict treatment decision making.
- A decision support tool to support biologic therapy withdrawal decisions has the most value in explaining the decision to children with nonsystemic juvenile idiopathic arthritis and their parents.

Juvenile idiopathic arthritis (JIA) is the most frequent chronic rheumatic condition in childhood, characterized by joint inflammation (lasting more than 6 wk) of unknown origin, with an onset before 16 y of age.^1^ The International League of Associations for Rheumatology distinguishes 7 JIA subtypes, which differ in clinical and laboratory features as well as in disease severity, with systemic JIA being both phenotypically and genetically different from the nonsystemic JIA subtypes.^2^ Uveitis is the most common comorbidity in JIA. It affects up to 20% of children with JIA and can lead to permanent vision loss.^1–3^

Conventional disease-modifying anti-rheumatic drugs (DMARDs) are currently the recommended first-line therapy in non-systemic JIA.^4^ Biologic DMARDs are, however, increasingly used, resulting in significant therapeutic benefit in terms of improved disease control. This limits long-term physical impairment and increases quality of life.^5^ While there is clear evidence-based guidance on when to start biologic therapy, guidance for pediatric rheumatologists on withdrawal after achieving clinically inactive disease (CID) is lacking.^4^

On one hand, timely withdrawal of biologic therapy has clear benefits. It avoids prolonged exposure of the child to minor adverse effects of biologic therapy, such as injection site reactions, and reduces the risk of more severe adverse events, including infections requiring hospitalization, and a potentially increased risk of malignancies.^6,7^ In addition, timely withdrawal can save health care resources.^8^ On the other hand, about 3 in 4 patients flare within 1 y after withdrawing biologic therapy,^9,10^ requiring either restarting the same biologic or switching to a different one. In their decision to withdraw biologic therapy, pediatric rheumatologists must assess the likelihood of successful withdrawal, defined as no flare within the first year, for each individual patient.

Risk factors associated with a higher risk of flare include having rheumatoid factor (RF)–positive polyarticular JIA and the duration of CID while on biologic therapy,^9^ whereas the absence of uveitis, prior treatment with tocilizumab, and early initiation of biologic therapy were associated with a lower risk of flare.^11^ Other factors, such as younger age at JIA diagnosis, male sex, antinuclear antigen positivity, disease duration, and delayed remission, were inconsistently identified as predictors of flare.^9,10^ Thus, while some evidence informs the assessment, decisions regarding biologic therapy withdrawal largely rely on the pediatric rheumatologist’s judgment of the child’s individual risk of flare.

In the Understanding Childhood Arthritis Network (UCAN) Canada-Netherlands consortium, we identified the need among pediatric rheumatologists for support in decision making regarding biologic therapy withdrawal. To improve understanding of how these decisions are currently made, we first identified patient, disease, and contextual factors that influence pediatric rheumatologists’ decision making.^12^ Second, we determined the relative importance of these factors to pediatric rheumatologists in withdrawal decision making in 2 studies.^13,14^

This article outlines the third step, addressing the need among pediatric rheumatologists for more decision support: the development of a decision support tool (DST). We used the findings of the previous studies to develop a multicriteria DST that could support pediatric rheumatologists in making biologic therapy withdrawal decisions. We also describe the evaluation of the DST in terms of its design, content, feasibility, and acceptance by end users to support decision making in clinical practice.

## Methods

The UCAN consortium has enabled 2 integrated multicenter prospective studies, UCAN CAN-DU and UCAN CURE, jointly aiming to transform the care and outcomes of children living with arthritis. The UCAN consortium includes all pediatric rheumatologists from all leading pediatric rheumatology centers in Canada (*n* = 68) and the Netherlands (*n* = 18). The development and evaluation of a DST for biologic therapy withdrawal is a key deliverable of the UCAN CURE project. The study team used multicriteria decision analysis (MCDA) as a framework to develop the DST. MCDA is often used to support decisions that require tradeoffs between multiple, often competing criteria.^15–17^ The development of the MCDA model followed the Best Practices Guidelines for MCDA of the Professional Society for Health Economics and Outcomes Research.^18,19^

The study consisted of 4 phases: 1) determining the scope and purpose of the DST, 2) developing the MCDA model, 3) designing the DST prototype, and 4) evaluating the DST prototype. The study received approval from the ethics committee of the University of Twente, the Netherlands (No. 200741, 210684, and 230768) and of the University of Calgary, Canada (REB19-0360).

### Phase 1: DST Scope and Purpose

The core team determined the scope and purpose of the DST iteratively. This team consisted of 5 experts in the design and analysis of health preference methods and MCDA and 4 pediatric rheumatologists. The core team held bimonthly virtual meetings throughout the 3-y study. In addition, input from the wider UCAN consortium was gathered through presentations at annual consortium meetings. Feedback received during these meetings was discussed by the core team meetings and integrated when considered valuable and within scope.

### Phase 2: Development of the MCDA Model

#### 2a. Selection of the criteria set for the MCDA

In MCDA, a criterion is used to compare and evaluate the options. These options represent the possible courses of action among which a decision must be made. In the management of JIA, the 2 options after CID is achieved are to continue or withdraw biologic therapy.

A complete set of criteria (patient, disease, and treatment characteristics that influence the decision to withdraw biologic therapy in children with JIA) was previously determined through a literature review and a focus group with pediatric rheumatologists from Canada.^12^ To be included in the MCDA model, all criteria in the set had to be relevant, nonoverlapping, and preferentially independent.^19^ To assess the extent to which the identified criteria met these requirements, all pediatric rheumatologists involved in the UCAN CAN-DU consortium from the Netherlands (*n* = 18) were invited to participate in a semi-structured interview. These interviews were also used to understand the current decision-making process and to explore initial ideas among users about the design of the DST. The interview guide is included in Appendix 1. Interviews were conducted via Microsoft Teams and audio recorded with participants’ permission. Deductive content analysis, using the criteria previously identified in a focus group,^13^ was performed within a pragmatic epistemological framework to summarize the findings.^20,21^ New information was coded inductively. Participants were sent a summary of the interview findings and reviewed it for accuracy. The core team reviewed the findings for relevance to the Canadian context and made minor adjustments.

#### 2b. Populating the MCDA model

Phase 2a resulted in the selection of 9 criteria deemed most relevant to the withdrawal decision (see Appendix 2). These criteria included characteristics of the child, the disease, and the treatment with biologic therapy. For each characteristic, the existing variation among children with JIA, the disease itself, or treatment characteristics influencing the withdrawal decision was described as levels.^19^ Some characteristics were measured on a continuous scale, such as the time in CID, and others on a dichotomous scale, such as whether a child prefers to continue or withdraw biologic therapy.

To estimate the likelihood of continuing or withdrawing biologic therapy based on patient, disease, and treatment characteristics, a clinical vignette study was conducted. A detailed description of this survey-based study is available elsewhere.^14^ This study produced a regression-based predictive model that estimates the likelihood of withdrawal based on pediatric rheumatologists’ stated preferences. For each patient profile (a combination of different levels for each criterion), the value of continuing biologic therapy was estimated by multiplying the regression coefficient for each level with the corresponding patient, disease, and treatment characteristics of the child (equation 1).

$$\begin{array}{c} U\;\mathrm{continue}=-1.61+0.55\mathrm{RS}+0.57\mathrm{RF}+1.36F\\ +0.87\mathrm{JD}+1.28U+0.75S+0.35\mathrm{TMJ}+1.09\mathrm{TF}\\ +1.85\mathrm{PPcontinue} \end{array}$$

Equation (1): The regression equation for biologic treatment decision making in children with nonsystemic JIA in CID. U_continue_ is the predicted value of continuing treatment with biologic therapy. Independent patient, disease, and treatment characteristics that increase the preference for continuation include RS = time between the start of biologic therapy and CID is between 6 and 12 mo (reference = <6 mo); RF+ = rheumatoid-positive JIA (reference = RF−); F = history of flares (reference = no history); JD = joint damage (reference = no JD); U = history of uveitis (reference = no U); S = spine involvement (reference = no S); TMJ = temporomandibular joint (TMJ) involvement (reference = no TMJ); TF = history of treatment failure (reference = no TF); and PPcontinue = a preference of parents and/or children to continue biologic therapy (reference = preference to withdraw). Regression coefficients were derived from a clinical vignette study and are provided in Appendix 3.

The predicted likelihood of biologic therapy withdrawal was calculated by multiplying the baseline probability of withdrawal, based on the time the child had been in CID, with the odds of withdrawal given the child’s specific patient, disease, and treatment characteristics (equation 2).

Equation (2): The regression model estimates the probability of biologic therapy withdrawal in children with nonsystemic JIA after achieving CID (time in months). Pwithdraw(CIDmonths) represents the predicted probability of withdrawal at a specific time (in months) after achieving CID. The model calculates this probability by multiplying the baseline probability of withdrawal—assuming no patient, disease, or treatment characteristics favoring continued treatment—with the odds of withdrawal, derived as (1 − odds of continued treatment), based on the individual profile of the child.

$$\begin{array}{c} P_{\mathrm{withdraw}}(\mathrm{CI}D_{\mathrm{months}})=-1.2861+0.7557\ln\\ (\mathrm{CI}D_{\mathrm{months}})\;*\left(1-\frac{e^{{\hat{U}}_{\mathrm{continue}}}}{1+e^{{\hat{U}}_{\mathrm{continue}}}}\right) \end{array}$$

### Phase 3: Design of the DST Prototype

The core team initially developed the DST offline and later worked with a Web developer (R.M.-M.) to program it into a Web-based prototype application using the open-source programming language PHP. Pediatric rheumatologists in the core team tested different versions of the DST before finalizing the prototype. The initial DST prototype consisted of 3 steps. First, the user provided information about the child’s situation across 9 patient, disease, and treatment criteria that influence the withdrawal decision. Second, the user was shown the likelihood that other pediatric rheumatologists would withdraw biologic therapy in that patient. Third, the user could adjust the relative importance of the criteria in the model and view the impact on the likelihood of withdrawal. Based on input from the pediatric rheumatologists in the core team, an additional page was added to allow users to include clinical information about the child that was not captured in the model. The final prototype DST is included in Appendix 4.

### Phase 4: Evaluation of the DST

Purposeful sampling was used to identify a subset of pediatric rheumatologists from the UCAN CAN-DU consortium who varied in age, gender, level of experience, and country (Canada and the Netherlands). Twelve pediatric rheumatologists were invited to participate as experts in 1 of 4 online 2-h focus group sessions organized to evaluate the DST. Informed consent was obtained for recording and transcription of the focus group in Microsoft Teams. Each session began with a brief introduction to the project and the session’s aim. Participants were then asked to access the DST using an individual login name and password. They browsed through the DST at their own pace. No further instructions were provided, unless participants requested support or asked questions. After approximately 10 min, or when participants indicated they were finished, each step of using the DST and potential barriers to completing the tool were discussed.

Using a semi-structured interview format, participants were asked for their opinions on the design and content of the DST, its acceptability, and its feasibility. Acceptability refers to their perception of the DST as beneficial and appropriate. This includes perceived advantages and disadvantages, potential applications, and the pediatric rheumatologist’s willingness to adopt and integrate the tool into practice. Feasibility refers to the extent to which the DST can be effectively implemented within existing clinical workflows, including practical considerations such as the time required for use and alignment with current clinical practice. In addition, participants were asked about the perceived benefits and harms of presenting specific outputs of the tool, such as a recommendation based on other pediatric rheumatologists’ opinions.

All participants in the focus group were asked to complete an online survey during or after the session to provide background characteristics, assess acceptance of the tool in clinical practice, and offer additional input on the topics discussed as well as any concerns, remarks, or feedback they had not voiced during the session. The survey is included in Appendix 5. The qualitative results from the focus groups and open-ended survey questions were analyzed using content analysis within a pragmatic framework^20,21^ for the variables feasibility and acceptance. Suggested improvements in content and design were implemented and tested in the subsequent focus group. The quantitative survey results were analyzed using descriptive statistics.

## Results

### Phase 1: The Scope and Purpose of the DST

Based on discussions within the core team and input from pediatric rheumatologists of the UCAN CAN-DU consortium, the core team decided that the primary objective of the DST would be to support the pediatric rheumatologists in their decision to withdraw or continue biologic therapy by providing insights into how the weight assigned to various decision criteria affect the overall decision. This approach was intended to increase transparency in decision making. In the DST, biologic therapy includes both biologics and Janus kinase/signal transducers and activators of transcription (JAK-STAT) inhibitors. During the project, the core team also decided to expand the DST to include a prediction based on the preferences of a larger group of pediatric rheumatologists, with the aim of improving consistency in decisions. To accommodate this, phase 2b was added to the project.

Other potential objectives of the DST discussed included facilitating the registration of withdrawal decisions made by pediatric rheumatologists and linking these decisions to clinical and disease characteristics. Another objective was to enhance the understanding of the conditions and circumstances under which pediatric rheumatologists choose to withdraw or continue biologic therapy.

### Phase 2a: Selection of the Criteria Set for the MCDA

Ten of 18 (56%) pediatric rheumatologists from the Netherlands involved in the UCAN CAN-DU consortium participated in the first round of interviews. The interviews revealed that pediatric rheumatologists make 3 different value judgments in their decision-making process. These considerations included 1) the perceived chance that the child will flare; 2) the level of flare risk that the pediatric rheumatologist, child, and parents are willing to accept to gain the benefits of withdrawing biologic therapy; and 3) the perceived chance that continuing treatment will reduce the risk of flare. Since all 3 considerations were reflected in the timing of the withdrawal decision, the core team decided to use time as dependent variable in the decision model.

#### The decision-making process in current clinical practice

None of the participants considered withdrawing biologic therapy if children had been in CID for less than 6 mo, except in cases of severe side effects. In such cases, the child was switched to a different biologic. Between 6 and 9 mo of CID, reasons for withdrawing biologic therapy included mild side effects, such as skin irritation, or a preference by the child and/or the parent, for example, due to fear of injections. The earliest withdrawal of therapy in a child without risk factors varied between 9 and 15 mo. In addition to previously identified risk factors, the decision to withdraw or continue biologic therapy also took into account the preferences of the child or parent. A figure illustrating the decision-making process is provided in Appendix 6.

#### Relevant criteria

The initial list of 18 patient-, treatment-, and disease-related characteristics (Appendix 7) was reduced to 9 criteria that were relevant, nonredundant, nonoverlapping, and preferentially independent. For example, the time to achieve CID (response time), the total time in CID since starting biologic therapy, and the total duration of biologic treatment were considered overlapping. Therefore, only response time and total time in CID were included. The age of the child was removed, as all participants agreed that although older children are more likely to have a complex disease and treatment history, age itself is not a decisive factor in the withdrawal decision. Access to care and medication was removed because they are specific to the Canadian context and therefore limit the tool’s generalizability. An important finding from the interviews was the influence of the child’s and/or parents’ willingness to withdraw biologic therapy on the decision-making process. This willingness is influenced by factors such as the burden of regular injections, minor side effects, and the (in)convenience of having a flare during certain life events, such as holidays or exams. Pediatric rheumatologists expressed differing views on how much this willingness should influence their decision making. Some stated they would never proceed with withdrawal if the child or parents were unwilling, while others said they would try to convince them if they believed withdrawal was safe. The final list of criteria is provided in Appendix 2.

### Phase 2b: Populating the MCDA Model

The final 9 patient, disease, and treatment characteristics included in the model, along with the relative importance of each criterion in the decision to withdraw biologic therapy, were determined through in a clinical vignette study. The relevant findings for this study are presented in Appendix 3.

### Phase 3: Design of a DST Prototype

The DST prototype (Appendix 4) included a general introduction at the top of the page and 4 separate pages (tabs). On page 1, users were presented with the 9 criteria identified in phase 2a. For each criterion, users indicated the level that best represented their patient. A slider was used to indicate time in CID (i.e., between 6 and 24 mo), while boxes were used for criteria with dichotomous outcomes (e.g., YES/NO or RF+/RF− disease). Completing this page was essential for the DST to calculate and display the output. Page 2 collected additional clinical information about the child for registration purposes. This information was, however, not used in the model. On page 3, users were shown a pie chart displaying the likelihood that pediatric rheumatologists would withdraw biologic therapy based on the patient information entered on page 1. Users were then asked to indicate their own decision (whether to withdraw or continue biologic therapy for this child). Page 4 displayed the relative importance of each criterion in the model on a numerical rating scale from 0 (not important) to 100 (very important), based on the findings from the clinical vignette study. The relative importance of the criteria was as follows: child and/or parents’ preference for biologic therapy withdrawal (21%), history of flares (16%), history of uveitis (15%), history of treatment failure with biologics (13%), history of joint damage (10%), history of spine involvement (9%), RF status of the patient (7%), response to biologic therapy (6%), and history of TMJ involvement (4%). Users were then instructed on how to adjust the relative importance of the criteria. Two outputs were displayed: a bar chart in the top right corner showing the adjusted relative importance normalized to a total of 100%, and a pie chart at the bottom showing the adjusted likelihood of withdrawal based on the updated model input.

### Phase 4: DST Evaluation

Eleven of the 12 (92%) pediatric rheumatologists from the UCAN CAN-DU consortium who were invited participated in a focus group session. Five of them had also taken part in the first round of interviews during phase 2a. One pediatric rheumatologist was unavailable to participate. Four focus group sessions were organized, each attended by 2 to 4 pediatric rheumatologists. Three focus groups were conducted in English and 1 in Dutch. Of the participants, 8 were female and 5 were based in Canada. The characteristics of the participating pediatric rheumatologists are presented in Table 1.

**Table 1:** Background Characteristics of Pediatric Rheumatologists that Participated in a Focus Group Session for Evaluating the Decision Support Tool

Characteristic	Value
Gender, *n* (%)	
Male	3 (27)
Female	8 (73)
Age, y, *n* (%)	
≤30	0 (0)
31–40	1 (9)
41–50	3 (27)
>50	7 (64)
Country in which they practice, *n* (%)	
The Netherlands	6 (55)
Canada	5 (45)
Primary practice center, *n* (%)	
Academic setting, university based	11 (100)
Years in practice since training, *n* (%)	
≤5	1 (9)
6–10	0 (0)
11–20	5 (45)
21–30	4 (36)
>30	1 (9)
Percentage of time allocated to clinical work, mean (range)	61 (7–80)

Five key themes emerged consistently across discussions within and between the focus groups. The condensed results from these sessions are presented in Table 2.

**Table 2:** Overview of the Findings during the Focus Groups and Actions Based on These Findings

Problem Type	Definition	Example	Revision
Design and formatting			
General layout and appeal	Comments that represented preferences for location, color, and/or formatting that influence the general look and feel of the DST	“Currently there are two sets of tabs, at the very top as well as the tab at the bottom. It’s a little bit confusing. I know that one tab is more instructional tab and the others are filling in the things. Is there any possibility of using arrows like next instead of using tab? Because it’s not as intuitive to have two sets of tabs.”	Suggestions that received broad support by the focus group participants were incorporated to the extent possible within the limitations of the programming language.
Instructive text, formatting	Comments that indicated preferences for formatting of instructive text	“I actually ignored them [the blue boxes with instructions at the top].”	Format changes were made to draw attention to essential instructive text at the top. Additional information (definitions) was moved to the bottom of the page.
Instructive text			
General instruction	Comments that address the need for instructive text regarding scope, purpose and use of the DST	“One question that I think is or one comment that I think is generic across this is I think it there needs to be umm, I guess some kind of front page to the tool so people understand what it's for.”	A page with instructive text was added to the DST. The instruction included scope and purpose of the DST and explicitly listed indications for which the DST was not applicable. Instructive text at the top of each page was optimized.
Criteria labels, descriptions, or levels	Comments that ask for changes to criteria labels, their descriptions, or the options pediatric rheumatologists could choose	“One more thing is response to treatment. So the way that it’s phrased, how long did it take your patient to achieve first response? I don’t know what that means. . . . I think you mean I think do you mean clinically inactive disease there?	Criteria labels, descriptions, or levels were changed to match the suggestions from the pediatric rheumatologists to reflect the intended meaning (descriptions) and clinical situation (levels) and then tested in the next focus group.
Use of terminology	Comments that indicated opinions regarding the need to define terminology to the extent that there is an unequivocal understanding among users	“I checked the Wallace criteria in literature. . . . It is actually quite straightforward and I think this really reflects what most physicians, at least pragmatically, probably not by ticking the boxes, but use in clinical practice.”	Terms were defined as narrow as possible to ensure a common understanding among pediatric rheumatologists. The term *treatment period* was omitted from the DST because its definition remained ambiguous throughout the study.
DST			
Criteria, relevance	Comments that questioned or confirmed the relevance of the criteria in the DST to the decision	“This last section, patient preference. I want to ask #9, have you ever had a patient who said OH, don’t stop the biologic. Don’t stop the biologic. I don’t want you to stop the biologic. I mean, maybe my patients are just unique, but everybody is like, when can I stop the medication?”	No changes were made to the criteria on the first page of the DST. For all criteria for which the argument was made that it may not be relevant, the opposite argument (to keep the criterion) was also made by focus group participants. Some criteria were removed from the second (optional) page to avoid overlap, and 1 criterion was added based on the findings during the focus group.
Comprehension of input: relative importance of decision criteria and adjustment	Comments indicating a (mis)understanding of the criteria importance and its adjustment	“Maybe a better word as opposed to importance is the ‘contribution of each one of these factors’ because you’re basically trying to give them a weighting right now, right? So it is really a contribution.”	All suggestions were implemented if they conveyed the intention of the task and/or addressed the significance of the criteria to the decision.
Comprehension of output: likelihood of withdrawal	Comments indicating a (mis)understanding of the pie chart which presented the likelihood of withdrawal	“But what I feel is difficult, is what is the read of this? What I read now of 74% versus 26%. So here [the pie chart on page 4] the colour says the stop or continue. That now says that this is ‘the decision.’ I just don't know if we can find a better text around it.”	All suggestions were implemented if they conveyed the intention of the output.
Use of the DST			
Use of the DST in clinical practice	Comments that expressed opinions, ideas, and/or preferences regarding the use of the DST	“But it does look promising. For example, if you discuss it with the patient, it becomes very clear, right? . . . I think it’s nice to show, if you have patients who are hesitant, that you can demonstrate. Look, your colleagues would also mostly support this, and it can help support a treatment plan.	Not applicable
Next steps	Comments and/or suggestions regarding further development of the DST	“And so, you know, like it if it’s to collect data to be able to say what does Quebec doctors do versus BC doctors? Yes, that's a useful thing. And that's how we'll sell it.”	Not applicable

DST, decision support tool.

### Theme 1: The Need to Precisely Define Terminology to Ensure Uniform Interpretation among All Users

The prototype DST used terms such as *clinically inactive disease* and *treatment period*. While some pediatric rheumatologists did not request definitions during testing, others immediately questioned the intended meaning of these terms. Discussions revealed that about half of the participants accepted a degree of ambiguity, viewing it as reflective of real-world clinical practice:

The purpose of this is to guide a clinical decision that has been made at the bedside and different clinicians might use slightly different criteria and definitions. So instead of getting stuck on the definition of a criteria, . . . try to minimally use these terms and just get the idea across that it is really a clinically motivated decision point.

In contrast, others emphasized the need for precise and specific definitions, arguing that consistent interpretation is essential for the DST to be used reliably across different users.

### Theme 2: The Need for Concise Instructions on How and When to Adjust the Relative Importance of Criteria in the Model

Participants generally understood the purpose of the page that allowed them to adjust the relative importance of the criteria. They noted that 1) different criteria can carry varying levels of importance in a decision, 2) this importance may differ between individuals, and 3) the ability to adjust the prior weights of the criteria in the model is a valuable feature of the DST. Several participants suggested using alternative wording to explain the concept of “relative importance.” According to the pediatric rheumatologists, the output itself could be best explained as the “relative contribution” of the child, treatment, and disease-related characteristics to the withdrawal decision.

Overall, participants felt comfortable using the sliders to adjust the relative importance of criteria. However, perspectives varied regarding the graph that displayed the normalized importance. Two pediatric rheumatologists explicitly expressed discomfort with this graph. Although they understood that recalculating absolute importance on a 100-point visual analog scale to a relative importance summing to 100% was necessary for the model and that this recalculation did not alter their input, they felt that the graphical output “no longer truly reflects how I really feel.” In contrast, other participants appreciated how this graph provided insight into the relative contribution of the different criteria within the decision model. Suggestions were made to remove the graph entirely or to make it less prominent in the DST, for example, by relocating it to the bottom of the page.

### Theme 3: The Need to Practice with the DST to Increase Trust in the MCDA Model Output

Initially, some pediatric rheumatologists found it challenging to fully understand the relationship between the input and the output of the DST. They described the tool as confusing, nonintuitive, and not self-explanatory. However, after further instruction and several rounds of practice, most participants became engaged and enthusiastic. One participant remarked:

I’ve managed to create a situation where 100% of the children continues to use biologic therapy. And if I then set certain factors to zero, such as erosions and flares, there are some percentages that would indeed stop. Yes, so you can do that, you see changes occurring there!

Based on the suggestions from the pediatric rheumatologists, the wording of the instructive text was revised. By the final focus group session, pediatric rheumatologists understood what they had to do.

Practice rounds included modifying the input (i.e., changing the characteristics of the child, disease, and/or treatment), adjusting the relative importance of decision criteria, and observing the resulting changes in the model’s output. After some practice, the face validity of the DST’s output was perceived as high, with a higher probability of withdrawal in more favorable clinical scenarios and with increased relative importance of favorable child, disease, and treatment characteristics. However, some Canadian pediatric rheumatologists expressed surprise at the relatively high likelihood that other pediatric rheumatologists would choose to withdraw biologic therapy. One participant noted,

What I notice is that I’ve entered several scenarios, and in some, because I often stop, there’s really a lot of stopping here. Almost always, the pie chart [circle diagram] leans towards stopping. I have one scenario where I thought they really can’t advise stopping, and still, 25% say to stop.

They attributed this to differences in disease management between the Netherlands and Canada.

### Theme 4: User Acceptance and Feasibility of the DST in Clinical Practice

Most participants responded positively to the way the DST supports decision making by explicitly outlining the steps they currently take implicitly when considering withdrawal of biologic therapy. In addition, they felt the output would be particularly valuable for pediatric rheumatologists who are less experienced in making these decisions, either in general or with specific biologic therapies, especially given that not all biologics are available simultaneously across different geographical regions.

Two pediatric rheumatologists, however, questioned the necessity of the DST and the relevance of incorporating others’ judgments into their own clinical practice. They commented that the tool primarily supports decisions in the same way they are already made in practice and that it allows adjustments to support the decision they already have in mind.

So, this decision, the decision aid basically it makes you feel more comfortable or less comfortable with what you're going to do because, but you can just work it however you want. Basically, based on your own prior preferences, which kind of negates using the tool altogether. Because you can override it.

This led them to question whether they would use the DST in their daily clinical practice.

At present, the DST was developed to support pediatric rheumatologists. They were very positive about its potential use in explaining their decisions to patients or in helping patients gain more confidence in the decision itself, by showing which criteria influence the decision and that other pediatric rheumatologists would likely make a similar decision. They also noted that the DST could be valuable in initiating discussions with the child and parents about biologic therapy withdrawal and in sharing responsibility for the decision with them. No concerns were raised regarding feasibility.

### Theme 5: Future Improvements to the DST

Several areas for further development of the DST were identified, although they were outside the scope of the current study. These included the following:

1. expanding the sample size for the clinical vignette study and using the DST to collect information from more pediatric rheumatologists to strengthen the evidence base for the current DST;
2. adding an option for users to adjust predictions based on the context in which the decision is made (i.e., country, province, access to different biologic therapies);
3. incorporating the level of evidence for the influence of each criterion, based on clinical data currently collected in UCAN; and
4. including a value clarification method to the DST to help patient and parents independently clarify their preferences for biologic therapy withdrawal, as the current use of a combined criterion does not account for potential differences between them.

### Survey Findings

The survey was completed by 10 of 11 focus group participants. Responses to open-text fields were incorporated into the themes above. The quantitative results indicated that all pediatric rheumatologists wanted to retain the option to adjust the relative importance of the criteria. Acceptability of the DST was high. Nine of 10 respondents indicated they would use the DST to support their decision whether to taper or withdraw a biologic in a child with JIA and to enhance scientific understanding of how preferences influence such decisions. Seven of 10 respondents wanted the option to save information for use in subsequent appointments. In terms of feasibility, 2 respondents were willing to spend 1 to 2 min using the DST, 6 were willing to spend 3 to 5 min, and 2 were willing to spend 5 to 10 min. Most participants (8 of 10) preferred the pie chart over a bar chart for presenting the overall results. The full survey results are presented in Appendix 8.

## Discussion

In this study, we developed and evaluated a DST to support biologic therapy withdrawal decisions in JIA using MCDA methods, aiming to facilitate decision making in the absence of clinical evidence and to improve consistency within and between pediatric rheumatologists. Overall, attitudes toward the DST and its use in clinical practice were positive. Perceived benefits included the structure it brings to the decision-making process, the opportunity to learn from peers, particularly valuable for less experienced pediatric rheumatologists, and its potential to involve patients and parents in the decision-making process. Suggestions from the focus groups regarding the language, content, and layout of the DST were incorporated. The most significant change following the evaluation was the removal of the term *treatment period*, as its definition remained ambiguous and the concept was unfamiliar and therefore difficult to explain to pediatric rheumatologists.

During the development of the DST, several difficult decisions had to be made. First, we needed to determine its scope and purpose. It was decided that the primary purpose would be to support pediatric rheumatologists in their decision to withdraw or continue biologic therapy. While preparatory work revealed the need for such support, opinions varied on what form it should take and whether a DST based solely on expert opinion would be useful. Ideally, most pediatric rheumatologists would prefer a DST based on clinical predictors, although the necessary clinical evidence to develop such a tool is currently lacking. As a result, additional aims of the DST were proposed. One was to use the DST to enhance the overall understanding of the conditions and circumstances under which pediatric rheumatologists currently choose to withdraw or continue biologic therapy. This insight could then inform updates to the model underlying the current DST. In the future, as more data are collected through actual use of the DST, the estimates for the likelihood of withdrawal would become more reliable, and it would be possible to incorporate contextual variables not currently included in the model, such as the country in which the decision is made. For example, it is known that in the Netherlands, biologic therapy is typically withdrawn earlier than in Canada, partly due to contextual factors such as access to biologic therapies. A third aim was to facilitate the registration of withdrawal decisions made by pediatric rheumatologists. When combined with information on whether the withdrawal attempt was successful, this would provide the data needed to develop an evidence-based DST. In general, our study showed a high willingness among participants to use the current DST for all 3 of these purposes.

Second, our initial intention was to develop an MCDA model in which pediatric rheumatologists, on an individual basis, would assign relative importance to various criteria and then be presented with the overall value of withdrawing versus continuing biologic therapy, based on their own judgments. During development, we abandoned this idea for 2 reasons. First, the decision to withdraw biologic therapy is binary: a longer duration in CID favors withdrawal, while the presence of many other patient, disease, and treatment factors favors continuation. Due to the imbalance between characteristics supporting withdrawal and those supporting continued treatment, achieving face validity of the MCDA model in clinical practice proved exceedingly challenging. Second, while an MCDA model based on pediatric rheumatologists’ own preferences can increase transparency and consistency of the decision on an individual level, it does not promote consistency of decisions between pediatric rheumatologists. Therefore, we decided to shift to a prediction model instead. To deepen our understanding of the likelihood of withdrawal given the presence of patient, disease, and treatment factors considered risk factors for a flare, we conducted the clinical vignette study.^14^ This allowed us to provide predictive information to pediatric rheumatologists and offer more concrete support for decision making. We retained the option to adjust the relative importance of model characteristics on an individual basis, as the decision model relied on group averages with wide confidence intervals. Also, allowing users to adjust the relative importance of different inputs increased transparency in the decision-making process.

Third, throughout the project, there was ongoing discussion about the tools intended for users, as both pediatric rheumatologists and children/parents influence the withdrawal decision, but their needs in decision support differ, resulting in potentially very different tools. For instance, pediatric rheumatologists tend to focus on disease characteristics and flare risk, while children and parents prioritize treatment burden in daily life.^13,22^ This influences the content of the value clarification exercise.

The term *decision support tool*, as used in this project, describes any system or resource that provides clinicians, patients, or other individuals with information and guidance to help them make informed decisions about health care. In contrast, a patient decision aid is aimed at patients and includes educational content regarding the disease and its treatment, the benefits and potential harms of withdrawal decisions, and a value clarification exercise.^23^

In this project, it was not feasible to develop both. Because the primary decision makers in this project were the pediatric rheumatologists, we chose to develop a DST. However, by integrating child and parental preferences as a criterion, we encouraged pediatric rheumatologists to discuss patient and parent preferences during clinical consultations, and we provide a structured way to explicitly incorporate them into the DST. Future iterations of the DST could include additional tools to further support shared decision making between pediatric rheumatologists, children with JIA, and their parents.

### Limitations

This study is subject to several limitations. First, the predictions of the likelihood of withdrawal and the relative importance of the criteria in the decision model were based on a relatively small number of pediatric rheumatologists from 2 countries with very different health care systems. As a result, the estimates in the current version of the DST have wide confidence intervals. While the ability for users to adjust the relative importance of the criteria on a case-by-case basis may help address this limitation, it comes at the cost of the tool’s ability to provide clear, uniform guidance for biologic therapy withdrawal decisions in JIA. As also recognized during the evaluation with users, future work should focus on collecting input from a broader group of pediatric rheumatologists working in diverse clinical settings.

Second, the pediatric rheumatologists who participated in the evaluation of the DST included some who were also involved in earlier development phases, and all participants worked in academic settings. They likely have more experience with withdrawal decision research than community-based pediatric rheumatologists do. However, since all pediatric rheumatologists in the Netherlands work in academic settings, this limitation regarding workplace applies only to the Canadian participants.

### Recommendations for Future Research

A next step in this project would be to implement the DST and to evaluate its feasibility, acceptance, and value in clinical practice. Data collected during real-world use, both regarding the relative importance assigned to criteria and the success of biologic therapy withdrawal, can be used to further refine the DST’s underlying statistical model. Successful implementation would require developing training materials and organizing practice sessions to familiarize users with the tool.

## Conclusion

The DST developed in this study was perceived as feasible and useful for clinical practice by most pediatric rheumatologists who evaluated it. Only relatively minor improvements were made to the design and instructions based on their feedback. The top priority for further development is to collect and incorporate clinical data on successful withdrawal decisions and to implement the tool in routine clinical practice. Data collected during actual use of this DST could be used to update and improve the underlying statistical model, both in terms of the judgments made and in identifying evidence-based risk factors that increase the likelihood of flares in children with non-systemic JIA.

## Supplemental Material

sj-docx-1-mpp-10.1177_23814683251364199 – Supplemental material for Development and Qualitative Evaluation of a Decision Support Tool for Withdrawal of Biologic Therapy in Nonsystemic Juvenile Idiopathic Arthritis Supplemental material, sj-docx-1-mpp-10.1177_23814683251364199 for Development and Qualitative Evaluation of a Decision Support Tool for Withdrawal of Biologic Therapy in Nonsystemic Juvenile Idiopathic Arthritis by Janine A. van Til, Michelle M. A. Kip, Robert Marinescu-Muster, Karin Groothuis-Oudshoorn, Gillian R. Currie, Susanne M. Benseler, Joost F. Swart, Sebastiaan J. Vastert, Nico Wulffraat, Rae S. M. Yeung, Deborah A. Marshall and Maarten J. IJzerman in MDM Policy & Practice

sj-docx-2-mpp-10.1177_23814683251364199 – Supplemental material for Development and Qualitative Evaluation of a Decision Support Tool for Withdrawal of Biologic Therapy in Nonsystemic Juvenile Idiopathic Arthritis Supplemental material, sj-docx-2-mpp-10.1177_23814683251364199 for Development and Qualitative Evaluation of a Decision Support Tool for Withdrawal of Biologic Therapy in Nonsystemic Juvenile Idiopathic Arthritis by Janine A. van Til, Michelle M. A. Kip, Robert Marinescu-Muster, Karin Groothuis-Oudshoorn, Gillian R. Currie, Susanne M. Benseler, Joost F. Swart, Sebastiaan J. Vastert, Nico Wulffraat, Rae S. M. Yeung, Deborah A. Marshall and Maarten J. IJzerman in MDM Policy & Practice

sj-docx-3-mpp-10.1177_23814683251364199 – Supplemental material for Development and Qualitative Evaluation of a Decision Support Tool for Withdrawal of Biologic Therapy in Nonsystemic Juvenile Idiopathic Arthritis Supplemental material, sj-docx-3-mpp-10.1177_23814683251364199 for Development and Qualitative Evaluation of a Decision Support Tool for Withdrawal of Biologic Therapy in Nonsystemic Juvenile Idiopathic Arthritis by Janine A. van Til, Michelle M. A. Kip, Robert Marinescu-Muster, Karin Groothuis-Oudshoorn, Gillian R. Currie, Susanne M. Benseler, Joost F. Swart, Sebastiaan J. Vastert, Nico Wulffraat, Rae S. M. Yeung, Deborah A. Marshall and Maarten J. IJzerman in MDM Policy & Practice

sj-docx-4-mpp-10.1177_23814683251364199 – Supplemental material for Development and Qualitative Evaluation of a Decision Support Tool for Withdrawal of Biologic Therapy in Nonsystemic Juvenile Idiopathic Arthritis Supplemental material, sj-docx-4-mpp-10.1177_23814683251364199 for Development and Qualitative Evaluation of a Decision Support Tool for Withdrawal of Biologic Therapy in Nonsystemic Juvenile Idiopathic Arthritis by Janine A. van Til, Michelle M. A. Kip, Robert Marinescu-Muster, Karin Groothuis-Oudshoorn, Gillian R. Currie, Susanne M. Benseler, Joost F. Swart, Sebastiaan J. Vastert, Nico Wulffraat, Rae S. M. Yeung, Deborah A. Marshall and Maarten J. IJzerman in MDM Policy & Practice

sj-docx-5-mpp-10.1177_23814683251364199 – Supplemental material for Development and Qualitative Evaluation of a Decision Support Tool for Withdrawal of Biologic Therapy in Nonsystemic Juvenile Idiopathic Arthritis Supplemental material, sj-docx-5-mpp-10.1177_23814683251364199 for Development and Qualitative Evaluation of a Decision Support Tool for Withdrawal of Biologic Therapy in Nonsystemic Juvenile Idiopathic Arthritis by Janine A. van Til, Michelle M. A. Kip, Robert Marinescu-Muster, Karin Groothuis-Oudshoorn, Gillian R. Currie, Susanne M. Benseler, Joost F. Swart, Sebastiaan J. Vastert, Nico Wulffraat, Rae S. M. Yeung, Deborah A. Marshall and Maarten J. IJzerman in MDM Policy & Practice

sj-docx-6-mpp-10.1177_23814683251364199 – Supplemental material for Development and Qualitative Evaluation of a Decision Support Tool for Withdrawal of Biologic Therapy in Nonsystemic Juvenile Idiopathic Arthritis Supplemental material, sj-docx-6-mpp-10.1177_23814683251364199 for Development and Qualitative Evaluation of a Decision Support Tool for Withdrawal of Biologic Therapy in Nonsystemic Juvenile Idiopathic Arthritis by Janine A. van Til, Michelle M. A. Kip, Robert Marinescu-Muster, Karin Groothuis-Oudshoorn, Gillian R. Currie, Susanne M. Benseler, Joost F. Swart, Sebastiaan J. Vastert, Nico Wulffraat, Rae S. M. Yeung, Deborah A. Marshall and Maarten J. IJzerman in MDM Policy & Practice

sj-docx-7-mpp-10.1177_23814683251364199 – Supplemental material for Development and Qualitative Evaluation of a Decision Support Tool for Withdrawal of Biologic Therapy in Nonsystemic Juvenile Idiopathic Arthritis Supplemental material, sj-docx-7-mpp-10.1177_23814683251364199 for Development and Qualitative Evaluation of a Decision Support Tool for Withdrawal of Biologic Therapy in Nonsystemic Juvenile Idiopathic Arthritis by Janine A. van Til, Michelle M. A. Kip, Robert Marinescu-Muster, Karin Groothuis-Oudshoorn, Gillian R. Currie, Susanne M. Benseler, Joost F. Swart, Sebastiaan J. Vastert, Nico Wulffraat, Rae S. M. Yeung, Deborah A. Marshall and Maarten J. IJzerman in MDM Policy & Practice

sj-docx-8-mpp-10.1177_23814683251364199 – Supplemental material for Development and Qualitative Evaluation of a Decision Support Tool for Withdrawal of Biologic Therapy in Nonsystemic Juvenile Idiopathic Arthritis Supplemental material, sj-docx-8-mpp-10.1177_23814683251364199 for Development and Qualitative Evaluation of a Decision Support Tool for Withdrawal of Biologic Therapy in Nonsystemic Juvenile Idiopathic Arthritis by Janine A. van Til, Michelle M. A. Kip, Robert Marinescu-Muster, Karin Groothuis-Oudshoorn, Gillian R. Currie, Susanne M. Benseler, Joost F. Swart, Sebastiaan J. Vastert, Nico Wulffraat, Rae S. M. Yeung, Deborah A. Marshall and Maarten J. IJzerman in MDM Policy & Practice
