# Supplementary material for: Development and Qualitative Evaluation of a Decision Support Tool for Withdrawal of Biologic Therapy in Nonsystemic Juvenile Idiopathic Arthritis
Source: MDM Policy Pract. 2025 Sep 29;10(2):23814683251364199. doi: 10.1177/23814683251364199 (PMC12480790; doi:10.1177/23814683251364199)
Supplement: sj-docx-1-mpp-10.1177_23814683251364199 – Supplemental material for Development and Qualitative Evaluation of a Decision Support Tool for Withdrawal of Biologic Therapy in Nonsystemic Juvenile Idiopathic Arthritis [file sj-docx-1-mpp-10.1177_23814683251364199.docx]

**Appendix 1. Interview Guide**

**Introduction:**

Based on the current state of evidence, it is hard to decide whether and when biological DMARDs, with or without concomitant MTX, can be withdrawn in children who have clinically inactive disease.

This study's objective is to develop a decision-making tool to support taper or discontinue treatment with biologic therapy by weighing various relevant considerations against each other.

**The objectives of this interview are:**

Objectives during the first round of interviews are to:

- Improve our understanding of the decision process regarding withdrawal of biologic therapy
- Complete a list of criteria, arguments or reasons, that influence your decision whether to withdraw biologic therapy

Open question: Can you describe your decision process regarding withdrawal of biologic therapy? Ask follow-up questions if relevant:

1. When would you first consider withdrawing biologic therapy?
2. Do you discontinue or taper biologic therapy?
3. Are there circumstances in which you would withdraw biologic therapy sooner than [answer question 1]?
4. Are there circumstances in which you would withdraw biologic therapy later than [answer question 1]?

In the table below, you will find a list of criteria that we currently believe play a role in the decision to taper or discontinue treatment with biologic therapy.

**List**

1. Age
2. JIA subtype [systemic; oligoarthritis; polyarthritis (RF negative/positive); psoriasis; enthesitis; undifferentiated]
3. Joint damage or erosive condition
4. Eye inflammation (uveitis)
5. Involvement of the spine or SI joint
6. Involvement of the temporomandibular joint
7. Previous flares of JIA
8. Patient has inflammatory bowel disease or psoriasis
9. Duration of conventional treatment
10. Treatment failure with biologicals
11. Time between initiation of biological treatment and achieving remission
12. Time in CID
13. Total time in CID since starting biologic therapy,
14. [Limited] Access to care
15. [Limited] Availability of medication
16. Child's personal circumstances
17. Burden of treatment for children and parents (injections)
18. Minor side effects of treatment

**Questions**:

We use the term 'criterion,' but you can also think of this term as a reason, a circumstance, or an argument that you would consider when deciding on withdrawal of biologic therapy.

1. Is the list complete (no items that influence your decision are missing)?
2. Are some of the criteria on the list redundant (i.e. do not help you to make a biologic therapy withdrawal decision when this criterion is present)?
3. What scale or classification do you use to assess the patient for each criterion? For example, age can be measured in years/months/days, joint damage can be classified as present or absent, or mild/moderate/severe.
4. How does each criterion on the list influence your decision to withdraw biologic therapy? When answering, consider the following sentence: "If the patient ..., then I am more/less inclined to choose to taper or discontinue treatment."
5. Can you think of situations, for each criterion separately or together, where you would always withdraw biologic therapy?
6. Can you think of situations, for each criterion separately or together, where you would never withdraw biologic therapy?
7. (In what way) do you trade the outcomes of the various criteria against each other? For instance, can outcomes compensate for each other [e.g., "If the patient has joint damage, I am more inclined to withdraw biologic therapy if they achieve remission within 3 months than if it takes longer"]?
8. Are some of the criteria related? For instance, are their outcomes influenced by the same underlying characteristic and/or circumstances?
9. Are some criteria overlapping? For instance, are they partly measuring the same thing?
10. Are some criteria or outcomes dominant? For instance, when a patient is RF-, you will consider outcomes on other criteria, but if the patient is RF+, you think everything else is irrelevant?
