# Supplementary material for: Development and Qualitative Evaluation of a Decision Support Tool for Withdrawal of Biologic Therapy in Nonsystemic Juvenile Idiopathic Arthritis
Source: MDM Policy Pract. 2025 Sep 29;10(2):23814683251364199. doi: 10.1177/23814683251364199 (PMC12480790; doi:10.1177/23814683251364199)
Supplement: sj-docx-2-mpp-10.1177_23814683251364199 – Supplemental material for Development and Qualitative Evaluation of a Decision Support Tool for Withdrawal of Biologic Therapy in Nonsystemic Juvenile Idiopathic Arthritis [file sj-docx-2-mpp-10.1177_23814683251364199.docx]

**Appendix 2. Criteria and Levels Included in the Clinical Vignette Study**

| Criterion | Description | Levels |
| --- | --- | --- |
| Time to reaching clinical inactive disease* | How long did it take for your patient to achieve clinically inactive disease? | 6 to 12 months |
|  |  | < 6 months |
| Rheumatoid factor test result | Is your patient rheumatoid factor negative or positive? | Positive [includes unknown] |
|  |  | Negative |
| Occurrence of disease flares** | Did your patient previously experience a disease flare** after attaining clinically inactive disease*? | Yes |
|  |  | No |
| Presence of joint damage | Did your patient experience damage to the cartilage or bone (clinically documented, X-ray, ultrasound or MRI)? | Yes |
|  |  | No |
| Presence of uveitis*** | Does your patient have a history of uveitis, which is currently in remission? Please know that treatment with biologics is always continued if uveitis is still active. | Yes |
|  |  | No |
| Presence of Spine involvement | Did your patient experience disease activity in the cervical or lumbar spine? | Yes |
|  |  | No |
| Presence of TMJ involvement | Did your patient experience disease activity in the temporomandibular joint (TMJ)? | Yes |
|  |  | No |
| Patient/parent preference for biologics withdrawal | What does your patient (and/or their parents) prefer in terms of disease management? | Taper/Stop treatment with biologics |
|  |  | Continue treatment with biologics |
| Failure on previous biologics | Did your patient have a (primary or secondary) treatment failure with a previous biologic? | Yes |
|  |  | No |

*Clinical remission on medication is defined according to the Wallace criteria, which include: no active arthritis; no fever, no rash, serositis, splenomegaly, or generalized lymphadenopathy attributable to JIA; no active uveitis; normal erythrocyte sedimentation rate or C-reactive protein level (Wallace et al., 2004)
**A flare is defined as any recurrence of disease manifestations after first attaining clinical remission

*** Uveitis is assumed to be in remission at the same time as the JIA.

TMJ = temporomandibular joint
