# Supplementary material for: Development and Qualitative Evaluation of a Decision Support Tool for Withdrawal of Biologic Therapy in Nonsystemic Juvenile Idiopathic Arthritis
Source: MDM Policy Pract. 2025 Sep 29;10(2):23814683251364199. doi: 10.1177/23814683251364199 (PMC12480790; doi:10.1177/23814683251364199)
Supplement: sj-docx-3-mpp-10.1177_23814683251364199 – Supplemental material for Development and Qualitative Evaluation of a Decision Support Tool for Withdrawal of Biologic Therapy in Nonsystemic Juvenile Idiopathic Arthritis [file sj-docx-3-mpp-10.1177_23814683251364199.docx]

**Appendix 3. Prediction Model derived from the Clinical Vignette Study**

|  |  | Part-worth regression coefficients | Attribute Importance |
| --- | --- | --- | --- |
| Response Time | <6 months* | 0,00 | 6% |
|  | 6-12 months | 0,55 |  |
| Rheumatoid Factor | Negative* | 0,00 | 7% |
|  | Positive | 0,57 |  |
| History of Flares | No* | 0,00 | 16% |
|  | Yes | 1,36 |  |
| History of Joint Damage | No* | 0,00 | 10% |
|  | Yes | 0,87 |  |
| History of Uveitis | No* | 0,00 | 15% |
|  | Yes | 1,28 |  |
| History of Spine Involvement | No* | 0,00 | 9% |
|  | Yes | 0,75 |  |
| History of TMJ involvement | No* | 0,00 | 4% |
|  | Yes | 0,36 |  |
| History of Treatment Failure with Biologics | No* | 0,00 | 13% |
|  | Yes | 1,09 |  |
| Child and Parents Preference | Withdraw* | 0,00 | 21% |
|  | Continue | 1,85 |  |

*Table 1. Odds ratios, betas, and importance weights of the different levels of the criteria in the model. * Indicates the reference level, which is the outcome for each criterion that is more likely to result in withdrawal of biologic therapy. TMJ = temporomandibular joint.*

**The predicted probability of withdrawing vs. continuing biologic therapy**

We estimated the probability of withdrawal at different moments in time after CID was achieved by presenting the pediatric rheumatologists with the vignette with the reference levels and asking them to indicate when (at which time in months) after achieving CID they would withdraw treatment in this child. We then fitted the data (equation 1).

$$P_{withdraw}\left( {CID}_{months} \right)=-1,2861+0.7557ln\left( {CID}_{months} \right)$$

*Equation 1. the logarithmic function used to estimate the probability of withdrawal after achieving remission. CIDmonths = time in months the JIA has been clinically inactive*

With nine criteria and two levels each, 512 (2^9^) possible patient profiles are possible. For each profile, the value of continuing biologic therapy was estimated by multiplying the regression coefficient for the level with the patient, disease, and treatment characteristics of the child (see equation 2).

Vcontinue=−1.61+0.55RS+0.57RF+1.36F+0.87JD+1.28U+0.75S+0.35TMJ+1.09TF+1.85PPcontinue

*Equation 2. Equation to calculate the utility of continuing treatment with biologics in children with non-systemic JIA. RS= time between start of biologic therapy and remission between 6 and 12 months; RF+=Rheumatoid Positive JIA; F=History of Flares; JD=Joint Damage; U=History of Uveitis; S=Spine involvement; TMJ = TMJ involvement; TF=History of Treatment Failure and PPcontinue= a preference of parents and/or children to continue biologic therapy.*

Then, the predicted probability that biologic therapy is continued is calculated using equation 3.

$$P_{continue}=\frac{e^{\hat{U}_{continue}}}{1+e^{\hat{U}_{continue}}}$$

*Equation 3. Equation to calculate the probability that treatment with biologics is continued in children with non-systemic JIA*

Finally, the probability of withdrawal is calculated by subtracting the probability of continuation based on the child characteristics from the baseline probability of withdrawal at different moments in time in an uncomplicated child with non-systemic JIA (equation 4).

$$P_{withdraw}\left( {CID}_{months} \right)=P_{withdraw}\left( {CID}_{months} \right)*{(1-P}_{continue})$$

*Equation 4. Equation to calculate the probability that treatment with biologics is withdrawn at different moments in time after achieving CID in children with non-systemic JIA.*

In the tool, pediatric rheumatologists can adjust the weights to see how weights influence the relative value of continuing vs. withdrawing biological therapy. When a weight is adjusted, the sum of weights always remains 1. Thus, if the weight of one of the factors increases, the relative weight of the other factors combined decreases with the same amount.
