# Supplementary figures and images for: Development and Qualitative Evaluation of a Decision Support Tool for Withdrawal of Biologic Therapy in Nonsystemic Juvenile Idiopathic Arthritis
Source: MDM Policy Pract. 2025 Sep 29;10(2):23814683251364199. doi: 10.1177/23814683251364199 (PMC12480790; doi:10.1177/23814683251364199)

**Appendix 4. Prototype DST**

Header


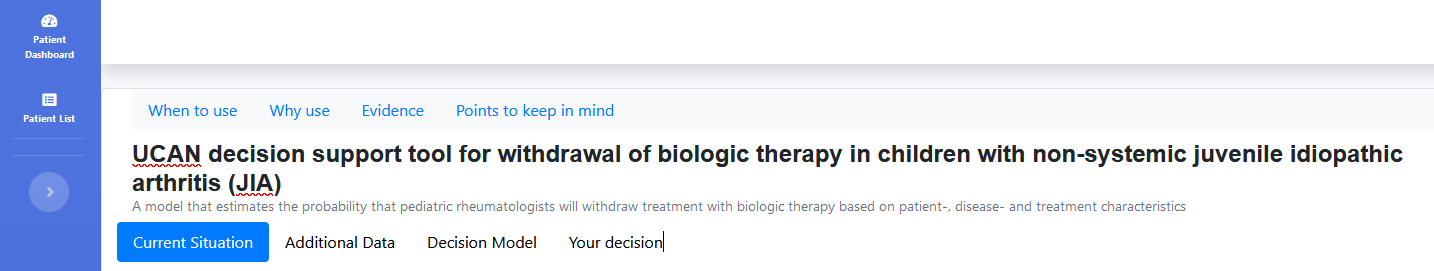


Page 1.


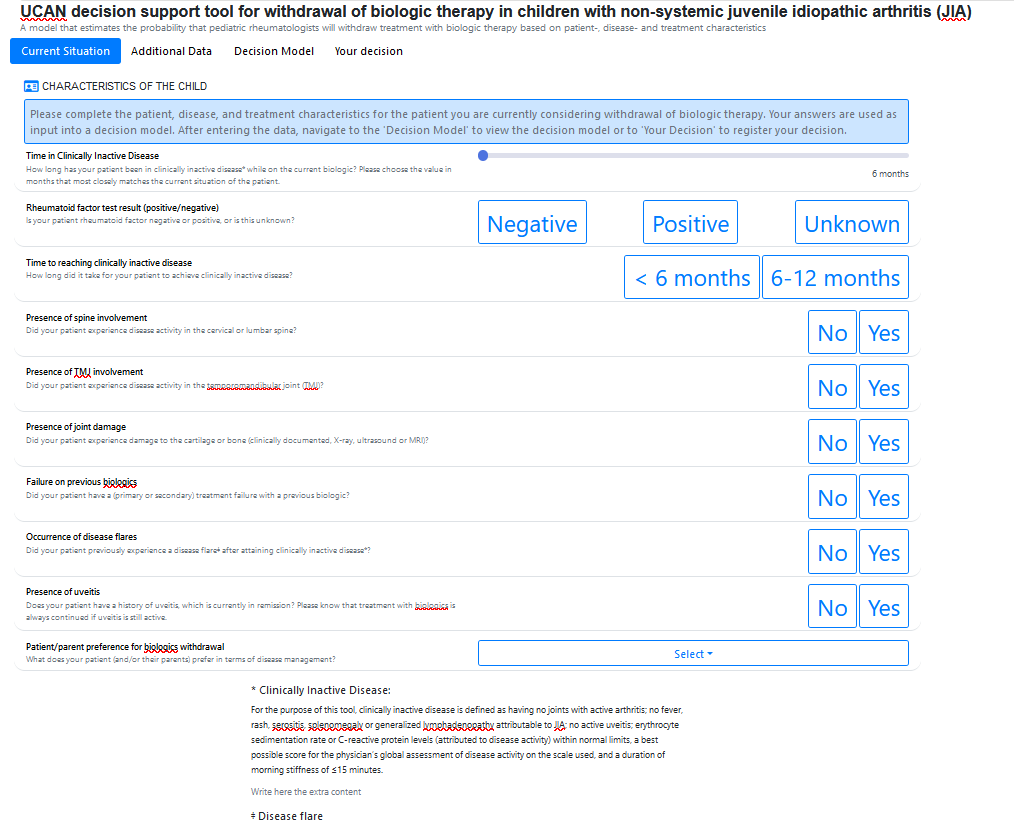


Page 2


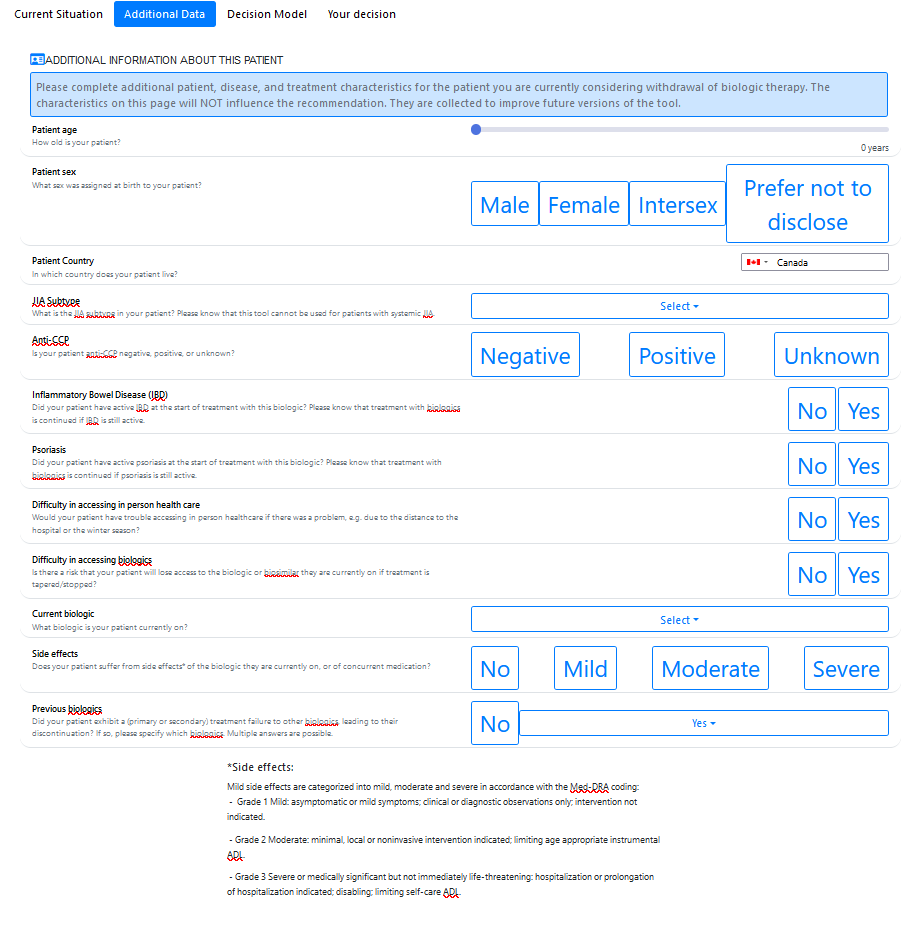


Page 3


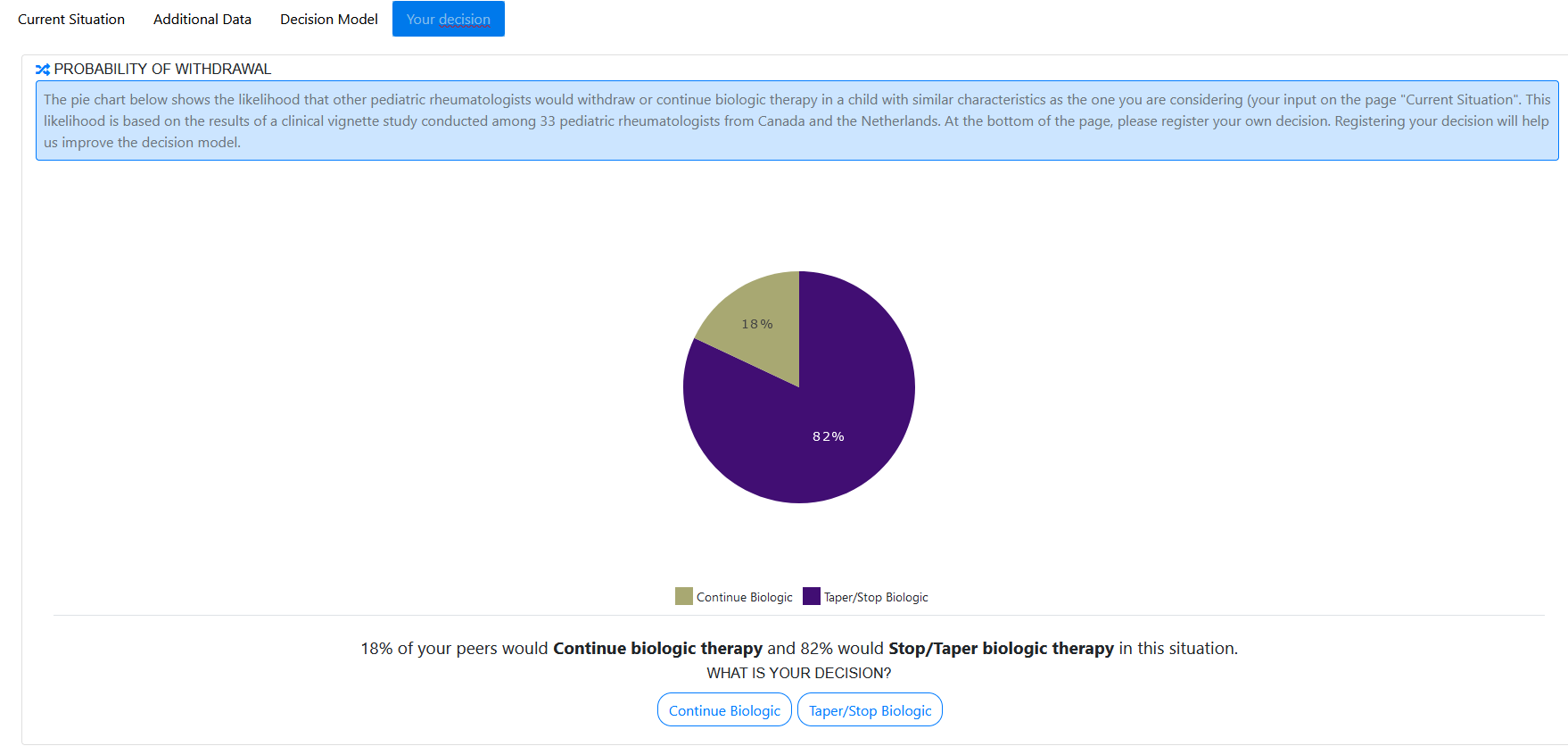


Page 4


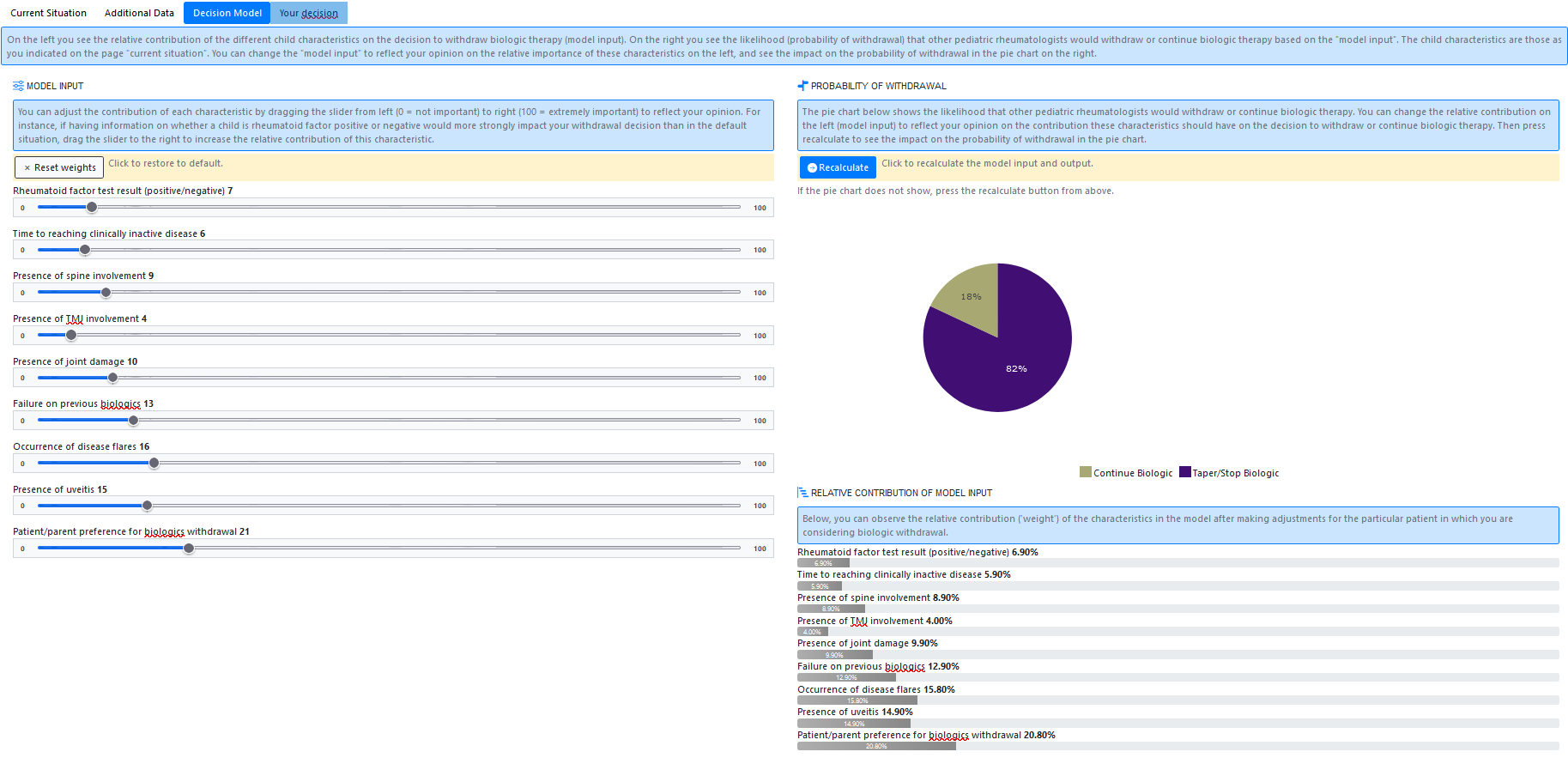

Supplement: sj-docx-4-mpp-10.1177_23814683251364199 – Supplemental material for Development and Qualitative Evaluation of a Decision Support Tool for Withdrawal of Biologic Therapy in Nonsystemic Juvenile Idiopathic Arthritis [file sj-docx-4-mpp-10.1177_23814683251364199.docx]
