# Supplementary material for: Development and Qualitative Evaluation of a Decision Support Tool for Withdrawal of Biologic Therapy in Nonsystemic Juvenile Idiopathic Arthritis
Source: MDM Policy Pract. 2025 Sep 29;10(2):23814683251364199. doi: 10.1177/23814683251364199 (PMC12480790; doi:10.1177/23814683251364199)
Supplement: sj-docx-5-mpp-10.1177_23814683251364199 – Supplemental material for Development and Qualitative Evaluation of a Decision Support Tool for Withdrawal of Biologic Therapy in Nonsystemic Juvenile Idiopathic Arthritis [file sj-docx-5-mpp-10.1177_23814683251364199.docx]

**Appendix 5. Survey Instrument**

**Start of Block: Introduction**

Q1.1 Dear pediatric rheumatologist,

You are being invited to participate in an expert panel to evaluate the design of a decision support tool for supporting decisions regarding biologic therapy withdrawal in patients with JIA.

This study is being initiated by a team of researchers from the University of Twente in the Netherlands and the University of Calgary in Canada, as part of the UCAN CAN-DU project. The aim of this study is to gain insight in the current decision process regarding tapering or stopping biologic therapy in children with JIA.

In this study, we aim to investigate your experiences with a concept decision support tool which is intended to support pediatric rheumatologists in decisions regarding withdrawing biologic therapy in JIA patients. The results of this survey will be used to finalize this decision support tool.

Your participation in this study is entirely voluntary and you can withdraw at any time. We believe there are no risks associated with this research study; however, as with any online related activity the risk of a data breach is always possible. We minimize any risks by storing data on secured servers and not capturing data that can be related to you as a person (IP address, e-mail address, traceable personal data such as date of birth). Ethical approval for this study was obtained from the University of Twente (no. 210684) and the University of Calgary (REB19-0360).

You can download a copy of this informed consent information here: (LINK TO IMPLIED CONSENT FORM).

If you have any questions regarding this study or this survey, you can contact xxx at xxx[@utwente.nl](mailto:j.a.vantil@utwente.nl) or xxx (xxx[@ucalgary.ca](mailto:currie@ucalgary.ca)).

 Thank you for considering to participate,
 Janine van Til
 Michelle Kip
 Gillian Currie
 Deborah Marshall
 Maarten IJzerman

Q1 Would you be willing to participate in this survey?

- Yes, I consent voluntarily to be a participant in this survey and understand that I can withdraw from the survey at any time, without having to give a reason. (1)
- No, I want to withdraw from participation in this survey. (2)

**End of Block: Introduction**

**Start of Block: Not participate**

Q2 You have indicated that you will not participate in the survey. To check whether people that are willing to participate are different from those that are not, we would like to ask you three short questions (age, sex, reason for not participating). Would you be willing to answer these three questions?

- Yes (1)
- No (2)

Q3
What is your gender?

- Male (1)
- Female (2)
- Non-binary / third gender (3)
- Prefer not to say (4)

Q4 What is your age?

- ≤30 (1)
- 31-40 (2)
- 41-50 (3)
- >50 (4)

Q5
What is the main reason to decline participation in this study?

- I am not a pediatric rheumatologist (1)
- I do not have the time to participate (2)
- I am not interested in the topic (3)
- I am not familiar with the topic (4)
- Other, namely (5) __________________________________________________

**End of Block: Not participate**

**Start of Block: 1. Open ended questions about the tools**

**Part 1: Evaluation of the decision support tool**
We developed a decision support tool, that distinguishes three main steps:

- First, users are asked to enter information about nine patient-, treatment- and disease characteristics. That information is then used to predict the preference of peer pediatric rheumatologists to withdraw biologics at different moments in time after clinically inactive disease is reached, in a child with similar characteristics as those that were entered in the tool.
- Then, users are asked to enter additional information on patient-, treatment- and disease characteristics. This information will allow improvements in future versions of the predictive model underlying the tool. Possible disadvantages are that filling in more details takes more time, while it currently does not affect the preference for withdrawing biologics.
- In the final step, users are allowed to adjust the relative importance of the abovementioned nine characteristics and observe the impact of their adjustments on the preference for continuing or withdrawal of biologics as indicated by peer pediatric rheumatologists.
  You can view and experiment with the tool through clicking with your **right mouse button** on this link**,** and choose '**Open link in new window**':

In the first part of this survey, we ask questions to learn more about your experiences while using the tool. We would like to know 1) what you like about the tool; 2) what you need to have changed in the tool to use it in clinical practice (must haves); and 3) what you wish could be changed in the tool (nice to haves) to improve your user experience.


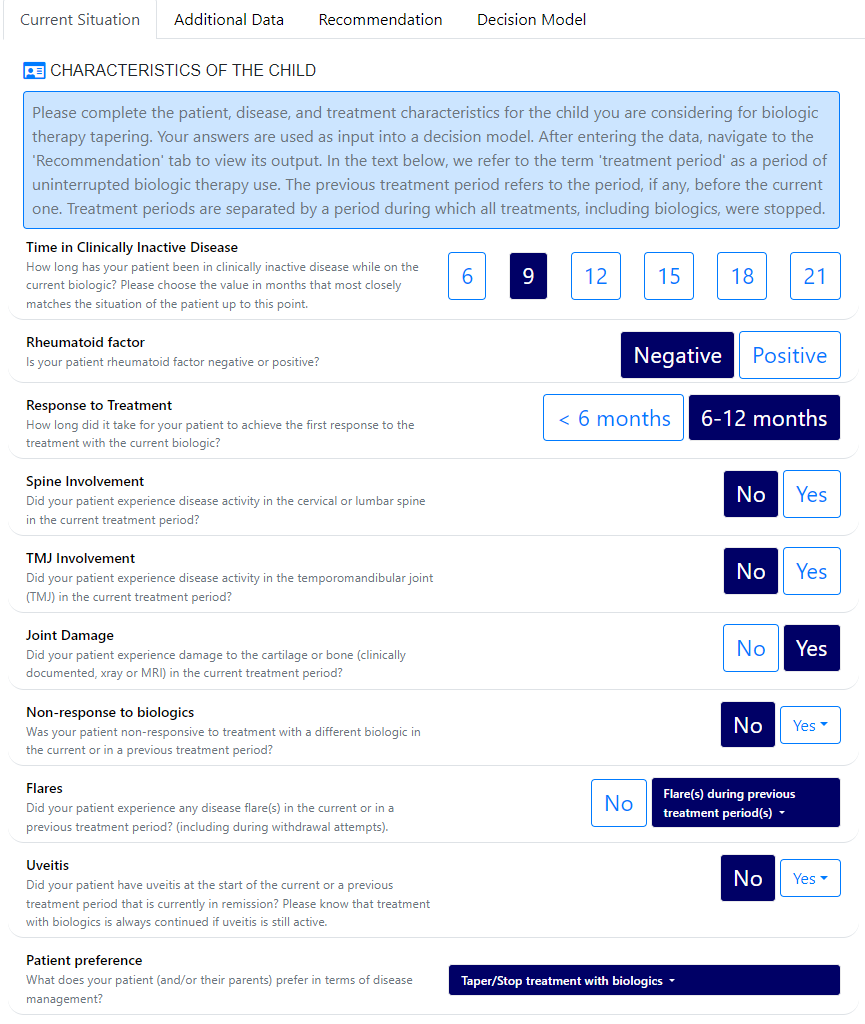


***Figure 1. Overview of the tool***

Q6
After you have entered the username and password, you are able to enter patient-, disease- and treatment characteristics, to view the outcome of the tool, and to manually adjust the weights assigned to the nine patient- disease- and treatment characteristics included on the first sheet. Take an example from your recent clinical practice to fill it in and to experiment with the tool.
Use the field below to describe what you like about the tool, for example regarding its design, content and/or its relevance for daily practice in its current form.

________________________________________________________________

________________________________________________________________

________________________________________________________________

________________________________________________________________

Q7 Which changes to the design or content of the tool are required to increase its relevance to clinical practice?

________________________________________________________________

________________________________________________________________

________________________________________________________________

________________________________________________________________

Q8 What would you like to see changed in the design or content of the tool, to improve your personal user experience?

________________________________________________________________

________________________________________________________________

________________________________________________________________

________________________________________________________________

Q9
After you have entered data, you see a pie chart which indicates the likelihood that your peers (pediatric rheumatologists that participated in a clinical vignette study) would either continue or taper/stop biologic therapy in a child with similar characteristics.


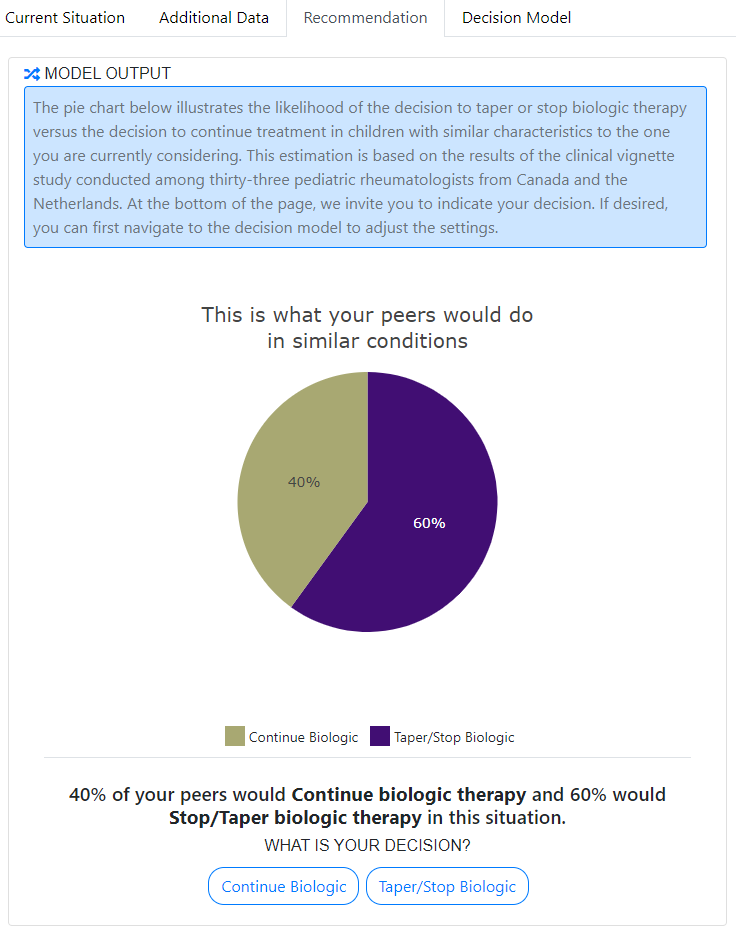


Q10 What are your thoughts about the benefits or positive outcomes of incorporating this information into the tool?

________________________________________________________________

________________________________________________________________

________________________________________________________________

Q11 What are your thoughts on the potential downsides or negative consequences of incorporating this information into the tool?

________________________________________________________________

________________________________________________________________

________________________________________________________________

Q12
Currently, the output of the tool is presented as a pie chart. Alternatively, the output can also be presented in a bar chart.
What is your preference for visualization of the output of the tool:

- A pie chart, because (1) __________________________________________________
- A bar chart, because (2) __________________________________________________
- No preference (3)
- Other, namely: (4) __________________________________________________

Q13
The tool uses the findings of the clinical vignette study that was performed within the UCAN CAN-DU project to calculate the output. This means that the collective input from pediatric rheumatologists was used to inform weights (i.e. the relative importance) of the nine patient-, disease- and treatment characteristics as shown on the first sheet of the tool, which thereby inform the current output of the tool. Currently, the tool allows you to manually adjust the weights of these patient-, disease- and treatment characteristics, and to input your individual weight.
 How do you feel about having the option to adjust the weights of the inputs in the model?

- I want to be able to adjust weights (the importance of criteria), because (1) __________________________________________________
- I don't need to able to adjust weights (the importance of criteria), because (2) __________________________________________________
- Don't know, because (3) __________________________________________________

**End of Block: 1. Open ended questions about the tools**

**Start of Block: Part 2. Potential Use of the Tool**

**Part 2: Potential use of a decision support tool**

A decision tool like this can be used for different purposes. In the following questions we ask you for your view about when such a tool could be used, and how much time using such a tool may take.

Q14 For which of the following purpose(s) do you think that a decision support tool **like the one** you just evaluated would be valuable (choose all that apply):

1. To support you in the decision whether to taper/withdraw a biologic in a child with JIA (1)
2. To inform the patient and/or parents of a child with JIA about the decision whether to taper/withdraw a biologic (2)
3. To involve the patient and/or parents of a child with JIA in the decision whether to taper/withdraw a biologic (3)
4. To offer insight in the congruence of your decisions across various children or at different points in time in the same child, to increase consistency between decisions (4)
5. To offer insight in the congruence of the decisions of different pediatric rheumatologists, to increase consistency between decisions (5)
6. None of the above (6)
7. Other, namely (7) __________________________________________________

Q15 Would you be willing to use the decision support tool, in its current form, for the purpose(s) that you indicated in question 14?

- Definitely not (1)
- Probably not (2)
- Might or might not (3)
- Probably yes (4)
- Definitely yes (5)

Q16 Would you be willing to use a next version of the tool, for the purpose(s) that you indicated, if it was aligned with your preferences regarding its design and content?

- Definitely not (1)
- Probably not (2)
- Might or might not (3)
- Probably yes (4)
- Definitely yes (5)

Q17 What is the maximum amount of time you would be able and willing to spend per patient on the use of the tool (this excludes discussing the results with the parents and/or child, if this is what you indicated above).

- 1-2 minutes (1)
- 3-5 minutes (2)
- 5-10 minutes (3)
- Other, please specify (4) __________________________________________________

Q18 Before we ask you to fill in some background characteristics about you as a person, are there any comments you have regarding the purpose, design and/or content of the tool that you have not been able to comment on in the questions so far? Please do so in this text field:

________________________________________________________________

________________________________________________________________

________________________________________________________________

________________________________________________________________

**End of Block: Part 2. Potential Use of the Tool**

**Start of Block: Part 3. Background Characteristics**

**Part 3. Respondent characteristics**
 Finally, we would like to ask you a few background characteristics about you as a participant, so we can report these in our study.

 Q19 What is your gender?

- Male (1)
- Female (2)
- Non-binary / third gender (3)
- Prefer not to say (4)

Q20 What is your age?

- ≤30 (1)
- 31-40 (2)
- 41-50 (3)
- >50 (4)

Q21 In which country do you practice medicine?

- Canada (1)
- the Netherlands (2) *which country do you practice medicine? = Canada*

Q22 In what province/territory do you practice medicine?

- Alberta (1)
- British Columbia (2)
- Manitoba (3)
- New Brunswick (4)
- Newfoundland and Labrador (5)
- Northwest Territories (6)
- Nova Scotia (7)
- Nunavut (8)
- Ontario (9)
- Prince Edward Island (10)
- Quebec (11)
- Saskatchewan (12)
- Yukon (13)

Q23 What is your primary practice setting?

- Academic setting, university based (1)
- Academic appointment but community-based practice (2)
- Solo community-based private practice (3)
- Group community-based private practice (4)
- Other (5) __________________________________________________

Q24 How many years have you been in practice since training?

- ≤5 years (1)
- 6-10 years (2)
- 11-20 years (3)
- 21-30 years (4)
- >30 years (5)

Q25 What percentage of your time do you allocate to clinical work?

________________________________________________________________

Q26 You have reached the end of this survey. Please use the text box below to provide any further suggestions or comments with regard to the decision support tool in general and/or with regard to the different versions of the tool:

________________________________________________________________

________________________________________________________________

________________________________________________________________

________________________________________________________________

________________________________________________________________

**End of Block: Part 3. Background Characteristics**
