# Supplementary material for: Development and Qualitative Evaluation of a Decision Support Tool for Withdrawal of Biologic Therapy in Nonsystemic Juvenile Idiopathic Arthritis
Source: MDM Policy Pract. 2025 Sep 29;10(2):23814683251364199. doi: 10.1177/23814683251364199 (PMC12480790; doi:10.1177/23814683251364199)
Supplement: sj-docx-6-mpp-10.1177_23814683251364199 – Supplemental material for Development and Qualitative Evaluation of a Decision Support Tool for Withdrawal of Biologic Therapy in Nonsystemic Juvenile Idiopathic Arthritis [file sj-docx-6-mpp-10.1177_23814683251364199.docx]

**Appendix 6. The decision process regarding withdrawal decisions derived from the interviews**
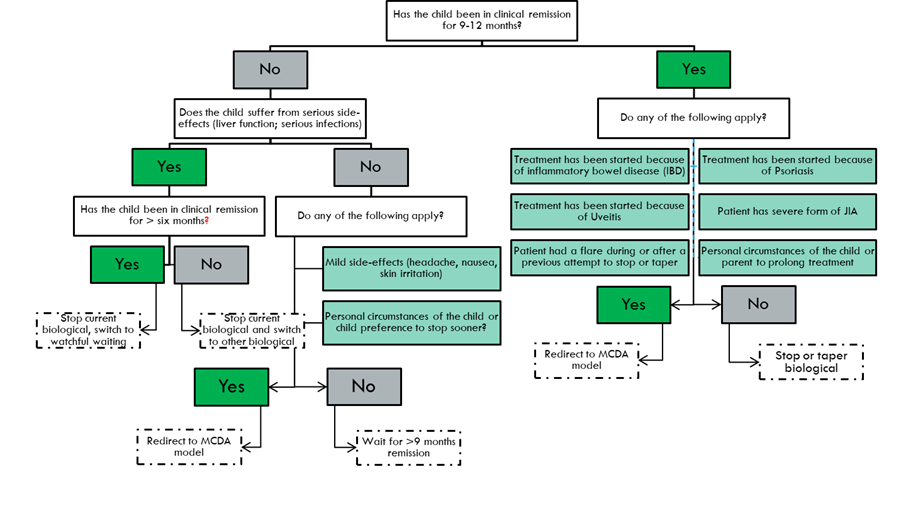


*Figure 1. Visualization of the two-arm decision path. The left branch of the decision tree contains criteria to withdraw biologic therapy sooner. The right branch lists the criteria to postpone a decision to withdraw treatment beyond 9-12 months.*
