# Supplementary material for: Development and Qualitative Evaluation of a Decision Support Tool for Withdrawal of Biologic Therapy in Nonsystemic Juvenile Idiopathic Arthritis
Source: MDM Policy Pract. 2025 Sep 29;10(2):23814683251364199. doi: 10.1177/23814683251364199 (PMC12480790; doi:10.1177/23814683251364199)
Supplement: sj-docx-7-mpp-10.1177_23814683251364199 – Supplemental material for Development and Qualitative Evaluation of a Decision Support Tool for Withdrawal of Biologic Therapy in Nonsystemic Juvenile Idiopathic Arthritis [file sj-docx-7-mpp-10.1177_23814683251364199.docx]

**Appendix 7. Initial Criteria**

The figure below shows the initial list of 18 patient-, treatment- and disease-related characteristics that resulted from the interviews. Each color represents a specific category of patient-, treatment- and disease-related characteristics: blue represents treatment characteristics, orange represents disease characteristics, light grey represents the preference of the child or parent to discontinue biologic therapy, yellow represents comorbidities, and green represents country-specific issues related to treatment and healthcare access. The months indicates the range of months that participants indicated to either advance or postpone the decision to withdraw biologic therapy when each characteristic was present.

# included in the clinical vignette study; *only relevant in the Canadian context
