# Supplementary material for: Development and Qualitative Evaluation of a Decision Support Tool for Withdrawal of Biologic Therapy in Nonsystemic Juvenile Idiopathic Arthritis
Source: MDM Policy Pract. 2025 Sep 29;10(2):23814683251364199. doi: 10.1177/23814683251364199 (PMC12480790; doi:10.1177/23814683251364199)
Supplement: sj-docx-8-mpp-10.1177_23814683251364199 – Supplemental material for Development and Qualitative Evaluation of a Decision Support Tool for Withdrawal of Biologic Therapy in Nonsystemic Juvenile Idiopathic Arthritis [file sj-docx-8-mpp-10.1177_23814683251364199.docx]

**Appendix 8. Survey results**

| **Results from multiple choice questions from survey** |  |
| --- | --- |
| Preference for visualization of the output of the tool (n, %):   - Pie chart - Bar chart - No preference | 8 (80%)  1 (10%)  1 (10%) |
| Preference about having the option to adjust the weights (the importance of criteria) (n, %):   - I want to be able to adjust weights - I don’t need to be able to adjust weights - Don’t know | 10 (100%)  0 (0%)  0 (0%) |
| Purposes for which the respondents would be willing to use the tool (n, %):   - For supporting them in the decision whether or not to taper/withdraw a biologic in a child with JIA - To increase transparency about how the decision about whether or not to taper/withdraw a biologic is made to children with JIA and/or their parents - To involve the patient and/or parents in the decision whether or not to taper/withdraw a biologic - To save information on patient-, disease-, and treatment characteristics and the extent to which they influence the decision to taper/withdraw at different moments in time. This will enable you (and the child/parents) to use that information at subsequent appointments, and thereby (potentially) increase consistency between decisions - To collect information on patient-, disease-, and treatment characteristics and how they influence tapering/withdrawal decisions, in order to increase our scientific understanding of how preferences influence tapering/withdrawal decisions (increase consistency between decision makers) - None of the above - Other, namely | 9 (90%)    8 (80%)    8 (80%)          7 (70%)        9 (90%)  0 (0%)  0 (0%) |
| What is the maximum amount of time you would be able and willing to spend per patient on the use of the tool (this excludes discussing the results with the parents and/or child, if this is what you indicated above) (n, %)   - 1-2 minutes - 3-5 minutes - 5-10 minutes - Other, please specify | 2 (20%)  6 (60%)  2 (20%)  0 (0%) |
| Would you be willing to use this decision support tool, for the purpose(s) that you indicated?   - Definitely not - Probably not - Might or might not - Probably yes - Definitely yes | 0 (0%)  0 (0%)  0 (0%)  6 (60%)  4 (40%) |
| Would you be willing to use the final version of the tool, for the purpose(s) that you indicated, if it was aligned with your preferences regarding its design and content?   - Definitely not - Probably not - Might or might not - Probably yes - Definitely yes | 0 (0%)  0 (0%)  0 (0%)  7 (70%)  3 (30%) |
